# Cardiovascular Calcification in Chronic Kidney Disease—Therapeutic Opportunities

**DOI:** 10.3390/toxins12030181

**Published:** 2020-03-14

**Authors:** Anika Himmelsbach, Carina Ciliox, Claudia Goettsch

**Affiliations:** Department of Internal Medicine I, Cardiology, Medical Faculty, RWTH, Aachen University, 52074 Aachen, Germany; ahimmelsbach@ukaachen.de (A.H.); cciliox@ukaachen.de (C.C.)

**Keywords:** chronic kidney disease, cardiovascular disease, vascular calcification, experimental rodent models

## Abstract

Patients with chronic kidney disease (CKD) are highly susceptible to cardiovascular (CV) complications, thus suffering from clinical manifestations such as heart failure and stroke. CV calcification greatly contributes to the increased CV risk in CKD patients. However, no clinically viable therapies towards treatment and prevention of CV calcification or early biomarkers have been approved to date, which is largely attributed to the asymptomatic progression of calcification and the dearth of high-resolution imaging techniques to detect early calcification prior to the ‘point of no return’. Clearly, new intervention and management strategies are essential to reduce CV risk factors in CKD patients. In experimental rodent models, novel promising therapeutic interventions demonstrate decreased CKD-induced calcification and prevent CV complications. Potential diagnostic markers such as the serum T50 assay, which demonstrates an association of serum calcification propensity with all-cause mortality and CV death in CKD patients, have been developed. This review provides an overview of the latest observations and evaluates the potential of these new interventions in relation to CV calcification in CKD patients. To this end, potential therapeutics have been analyzed, and their properties compared via experimental rodent models, human clinical trials, and meta-analyses.

## 1. Introduction

Clinically, interaction between organs is of growing relevance given the increasing number of elderly patients with many comorbidities and the recognition that such comorbidities not only influence the clinical course of a given disease and its prognosis, but also affect treatment options and therapeutic success [1]. To exemplify, impaired kidney function associates with poor outcome mainly due to a high burden of cardiovascular (CV) comorbidity, with its manifestations of ischemic heart disease, heart failure, or CV death—a major public health burden in developed countries [2]. Patients with chronic kidney disease (CKD) exhibit a more than four-fold (CKD stage > 3) higher CV risk compared to the non-CKD cohort [2]. Traditional strategies to reduce this risk are largely ineffective in CKD and end-stage renal disease (ESRD) patients, underscoring the importance of non-traditional CKD-specific CV risk factors that are hitherto unknown [2]. In the general population, classical atherosclerotic endpoints such as stroke or myocardial infarction are the dominant cause of death. Importantly, CKD patients mostly die from sudden cardiac death or ischemic heart disease due to premature vascular and cardiac aging [2]. 

CKD impairs the removal of harmful substances from the body. Therefore dialysis therapy is required to supplant the most important functions of the kidney. During dialysis, waste products like uremic toxins and excess salts and liquids are discharged via diffusion through a semipermeable dialysis membrane. Because of the small pore size of currently used dialysis membranes, protein-bound uremic toxins cannot be filtered during dialysis [3,4], leading to their presence in the blood of CKD patients, which might play a role in the development of cardiovascular disease (CVD). The protein-bound uremic toxin indoxyl sulfate is associated with CV death, and its levels correlate positively with CV calcification [5]. Thus, the existent dialysis therapy remains insufficient, which may explain the poor prognosis of ESRD patients [3]. 

**Figure 1:**
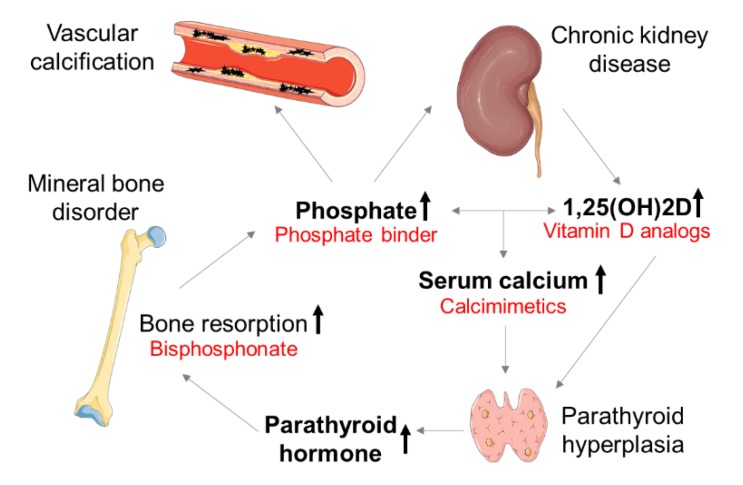
Pathogenesis of chronic kidney disease–mineral bone disorder (CKD–MBD). Targets for therapeutic strategies are written in red; 1,25(OH)2D: 1,25-dihydroxycholecalciferol (calcitriol). The figure was partially created using Servier Medical Art, licensed under a Creative Commons Attribution 3.0 Unported License. Black arrows indicate an increase.

These patients suffer from abnormalities in mineral metabolism, caused by an imbalance of calcification promoters (e.g., calcium and phosphate) and inhibitors (e.g., matrix Gla protein (MGP) and fetuin-A) [6] and termed ‘mineral bone disorder’ (MBD). The interconnection of phosphate, calcium, 1,25-dihydroxycholecalciferol (1,25(OH)2D), and fibroblast growth factor 23 (FGF-23) affects the kidney–parathyroid gland–bone axis [7]. In early CKD stages, physiological phosphate serum levels can be sustained. Renal phosphate is restricted by a decreasing glomerular filtration rate (GFR), causing hyperphosphatemia—a major challenge in CKD–MBD. In response to high serum phosphate, osteoblasts produce FGF-23, which inhibits 1,25(OH)2D production. Deficiency in 1,25(OH)2D lowers the serum calcium levels that stimulate the parathyroid gland to produce parathyroid hormone (PTH). The secondary hyperparathyroidism (sHPT) induces calcium efflux from the bone, leading to low bone mineral density. Vitamin D analogs and calcimimetics are used to suppress PTH. Bisphosphonates inhibit osteoclast activity and are applied to treat the dysregulated bone metabolism in CKD–MBD (Figure 1) [8]. Alterations of the bone mineral density are associated with the progression of aortic calcification in women but not in men [9]. Especially, postmenopausal women exhibit an increased risk for CV events [10]. The International Society of Nephrology (ISN) recommends frequent monitoring of serum levels of calcium, phosphate, and PTH, starting in CKD stage 3 patients [11]. If necessary, patients should be treated to maintain an age-appropriate physiological range of serum parameters.

**Figure 2:**
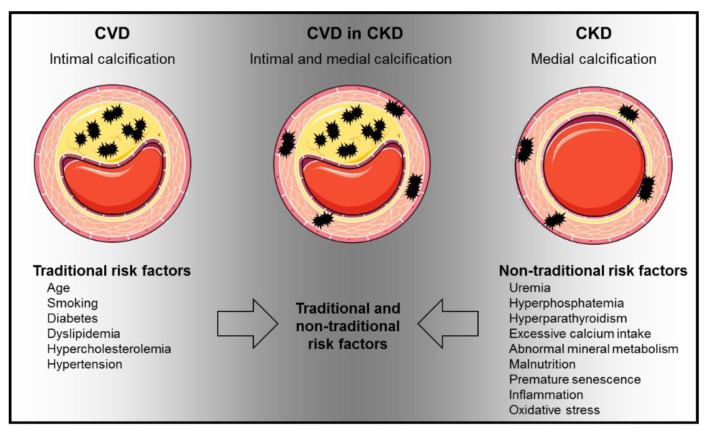
Traditional and non-traditional CVD risk factors affect uremia-induced calcification. Calcification in CKD can result within the tunica intima and tunica media. CVD, cardiovascular disease; The figure was partially created using Servier Medical Art, licensed under a Creative Commons Attribution 3.0 Unported License. Arrows indicate risk factors, which are present in CKD patients suffering from CVD.

Modifications in the circulation as well as in the myocardium are crucially involved in the increased CV risk in CKD patients. However, both the mediators and the underlying molecular mechanisms remain largely unexplored [12]. CV calcification—both in the tunica intima and in the media—is massively increased in CKD patients and is an independent risk factor for CV morbidity and mortality [13]. CV calcification could be one of the key mechanisms leading to increased CVD in CKD. CV calcification results from active cellular processes in which smooth muscle cells undergo phenotypic changes to build a mineralized matrix [14]. This process is supported by an imbalance of promoters and inhibitors of calcification, which promotes calcium and phosphate precipitation [15,16]. CKD patients with CV risk suffer from mineral deposits in the tunica media (arteriosclerosis) and tunica intima (atherosclerosis). The extent of CV calcification depends on the CKD stage [16,17]. Both traditional and non-traditional risk factors of CV calcification lead to the manifestation of CVD in CKD (Figure 2) [18,19]. 

Current treatments induce no adequate reduction of CV calcification in CKD, rendering the identification and development of promising therapeutic targets essential. Experimental rodent CKD models proffer novel promising treatments; for example, the hexasodium salt of myo-inositol hexaphosphate SNF472 has been suggested as a potent ectopic calcification inhibitor both in vitro and in vivo [20,21]. Recent studies suggest the peroxisome proliferator-activated receptor-gamma (PPARγ) and the mineralocorticoid receptor (MR) as novel molecular targets for CV complications in CKD. This review focuses on new potential treatments and compares their benefits in non-transgenic animal models with those in human clinical trials and meta-analyses. 

## 2. Animal Models of CKD

**Figure 3:**
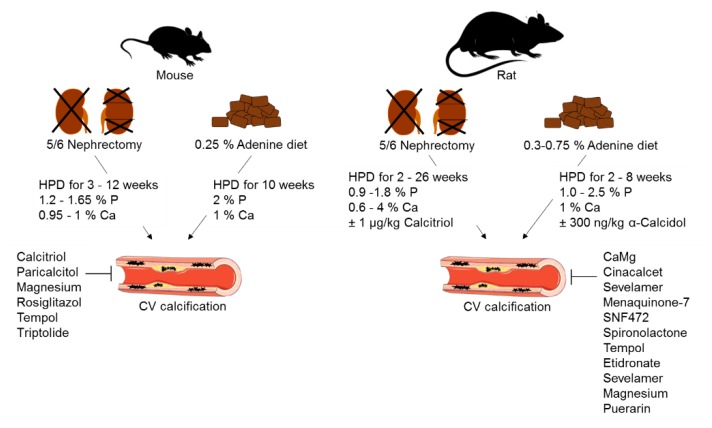
Schematic presentation of rodent non-transgenic animal models of cardiovascular calcification in CKD. HFD: high-phosphate diet; CV: cardiovascular; P: phosphate; Ca: calcium. The figure was partially created using Servier Medical Art, licensed under a Creative Commons Attribution 3.0 Unported License. Arrows indicate CV calcification induced by 5/6 nephrectomy and 0.25 % adenine diet. Fork indicates kidney areas, which are removed during 5/6 nephrectomy.

Animal models invariably provide valuable insights into the molecular mechanisms of diseases and their underlying pathology. However, none of the prevailing models reproduces the complexity of CVD in CKD [22]. While few non-transgenic rodent models are employed to study CV calcification in CKD [23], one such variant method is the reduction of renal mass via nephrectomy (Figure 3). Five-sixths nephrectomy is limited by the variability in CV calcification, the high mortality rate in patients with advanced CKD, and the necessity for surgery as an irreversible method [24]. Administration of dietary adenine is another strategy to initiate CKD in animal models (Figure 3); adenine is transformed to 2,8-hydroxyadenine, which precipitates in the urinary tract due to its low water solubility [25]. This causes nephrotoxicity, which is similar to clinical CKD [24]. The main disadvantage of this model is the weight loss of the animals due to reduced food intake. The adenine model is a reversible CKD model, because there is no need for surgery, which eases its implementation and handling. As neither five-sixths nephrectomy nor adenine diet alone initiate CV calcification, either high-phosphate or high-fat diet are used as a second trigger (Figure 3) [26]. Both models show similarities—hyperphosphatemia, increased plasma creatinine, and enhanced blood urea nitrogen, but the CV calcification outcome is not consistent. The reasons for this are differences in trial times and the high variability in diet phosphate and calcium concentrations [24]. The sensitivity to CV calcification also depends on the genetic background, age, and gender of the animals [27]. Female mice show higher susceptibility to CV calcification than males [28], which is the opposite to what observed in humans, wherein men tend to have higher average coronary artery calcium scores than women [29]. This might suggest that the hormone status affects vascular calcification and should be considered when planning experiments. CV calcification variabilities in CKD are mostly seen in mice but tend to be strain-dependent [22]. The most commonly used mouse strain is C57Bl/6, which is resistant to the development of hypertension, glomerulosclerosis, and proteinuria. In addition, it shows decreased activity in the renin–angiotensin–aldosterone system, which is important for fibrosis development after five-sixths nephrectomy [27,30]. In summary, compromises have to be made in choosing the right CKD animal model. Therefore, it is essential to agree on standards using rodent models within the CVD–CKD research field. 

## 3. Therapeutic Concepts of CV Calcification in CKD

### 3.1. Phosphate Binder

Hyperphosphatemia is a major clinical challenge in CKD–MBD. Phosphate binders (PB) are classified into calcium-based PB (CBB; e.g., calcium acetate, calcium carbonate) and non-calcium-based PB (e.g., sevelamer, lanthanum). The administration of PB reduces serum phosphate levels, thereby improving hyperphosphatemia in CKD patients. In two independent experimental CKD models, treatment with sevelamer attenuated vascular calcification (Table 1) [31,32]. The PB calcium acetate/magnesium carbonate (CaMg) reduced CV calcification without affecting bone mineral density in adenine-induced CKD rats (Table 1) [33]. 

In CKD patients, a meta-analysis of eight different PB (sevelamer, lanthanum, iron, calcium, colestilan, bixalomer, nicotinic acid, magnesium) showed that the PB reduced serum phosphate levels compared to placebo controls, but had no effect on all-cause mortality and CV events [34]. Another systematic review and meta-analysis revealed decreased all-cause mortality by non-calcium-based PB, compared to CBB in CKD patients [35]. A Cochrane systematic review and meta-analysis of randomized clinical trials (RCT) showed that sevelamer compared to CBB decreased all-cause mortality in ESRD patients [36], while sevelamer had no effect on CV mortality [37]. 

Based on these findings, the Kidney Disease: Improving Global Outcomes (KDIGO) 2017 guideline recommends PB treatment for progressively elevated phosphate and a restriction of CBB treatment [11], with a limited dietary phosphate intake [11]. Given a lack of evidence that PB reduce all-cause mortality, longer placebo-controlled trials are required. It also remains uncertain to which extent pre-dialysis patients would benefit from PB treatment, since adverse effects like nausea, constipation, diarrhea, and abdominal pain are reported [34].

### 3.2. Calcimimetics

Calcimimetics act on the calcium-sensing receptor and increase its sensitivity to calcium, thereby lowering the PTH level as a result of the feedback mechanism. Two generations of calcimimetics have been developed, the first of which—calcimimetic cinacalcet—is taken orally once daily. The second generation—calcimimetic etecalcetide—is applied intravenously three times per week after hemodialysis (HD) sessions [39]. 

In an experimental CKD model of adenine-fed rats, cinacalcet ameliorated aortic calcification (Table 1) [38]. The prospective RCTs EVOLVE and ADVANCE treated HD patients with sHPT daily with 30 to 180 mg cinacalcet [40,41]. In the ADVANCE trial, patients additionally received a low-dose vitamin D therapy. Cinacalcet reduced the progression of aortic valve calcification compared to the vitamin D control group, while it had no effect on aortic calcification [41]. Similar results were found in the EVOLVE trial. In both trials, cinacalcet bore no effect on all-cause mortality and CV event rate [40,41]. A meta-analysis of RCTs considering (pre)-dialysis patients and kidney transplant recipients (KTR) revealed that cinacalcet had no effect on all-cause mortality [42]. An observational study confirmed that cinacalcet is not associated with all-cause mortality but is related to reduced CV events [43]. A variety of adverse effects like diarrhea, hypocalcemia, and nausea have been reported [44]. While calcimimetics are quite effective in lowering serum PTH, the effect on all-cause mortality, CV risk, and calcification is uncertain [37]. Especially in pre-dialysis patients, further studies focusing on clinical rather than biochemical outcomes are needed. 

## 4. Novel Therapeutic Strategies—from Experimental Models to the Clinic

### 4.1. Bisphosphonates

Bisphosphonates, also known as pyrophosphate analogs, are antiresorptive drugs that are administered to treat diseases with high-turnover bone resorption, like osteoporosis, Paget’s disease, and multiple myeloma. In CKD–MBD, they are applied to treat the dysregulated bone metabolism [8]. Bisphosphonates inhibit osteoclast activity. There are two groups of bisphosphonates, with different nitrogen content. Non-nitrogen-containing bisphosphonates (e.g., etidronate) cause osteoclast apoptosis, while nitrogen-containing equivalents (e.g., alendronate; pamidronate) inhibit osteoclast activity. Nitrogen-containing bisphosphonates show 10–10,000 times increased potency in inhibiting bone resorption [45]. Bisphosphonate-associated nephrotoxicity has been reported [46,47]. Especially, intravenously applied bisphosphonates can cause acute kidney injury [47,48,49]. Therefore, doses and treatment period has to be adjusted in patients with pre-existing CKD [46]. Other known side effects are focal segmental glomerulosclerosis, hypocalcemia, and pathological fractures like bisphosphonate-related osteonecrosis of the jaw [46,50]. Still, bisphosphonates are in generally well tolerated, and severe side effects are rare [50,51,52]. The mechanisms of action and pharmacokinetics of bisphosphonates have recently been reviewed [53]. 

Etidronate reduced aortic calcification in five-sixths nephrectomy-induced CKD rats (Table 2), as well as in HD and CKD patients [54,55]. Alendronate did not alter aortic calcification in CKD stages 3 and 4 [56]. These results suggest that the nitrogen content of bisphosphonates may affect the potency of bisphosphonates to alter CV calcification. A systematic review summarized 20 performed trials and illustrated contrasting results of the existing bisphosphonate studies [55]. In CKD patients, coronary artery calcification (CAC) and aortic calcification were increased after 12–24 months of bisphosphonate treatment. In a non-CKD cohort of postmenopausal osteoporotic women, intima–media thickening was reduced under bisphosphonate therapy [55]. Evidence remains unclear regarding the effect on arterial stiffness and atherosclerotic plaques in humans. In a retrospective study, female CKD patients had a 22% reduced risk for all-cause mortality when treated with bisphosphonates. However, there was no benefit regarding CV mortality [57]. In different cohorts, beneficial effects were found on arterial calcification, but not on arterial stiffness. CV events were not improved by bisphosphonate therapy [58]. Due to the small amount of studies performed in CKD patients, evidence for a beneficial effect of bisphosphonates on vascular calcification in CKD–MBD is still unclear.

A novel strategy to alter osteoclast activity is the use of a neutralizing antibody against receptor activator of NFκB-ligand (RANKL), called denosumab, which inhibits bone resorption and reduces fracture risk [59]. RANKL is crucial for proper osteoclast function [60] and was shown to promote vascular calcification in vitro and in vivo [61,62]. In contrast to bisphosphonates, denosumab is not eliminated by the kidney [63] and appeared to be safe in HD patients. Nevertheless, a recent study revealed a denosumab-associated increased risk of renal function decline in male patients, patients with renal insufficiency, and patients with acute kidney injury [63]. In HD patients, neither alendronate nor denosumab treatment improved vascular function and CAC score [63]. 

Clinically and in animal models, there is an association between osteoporosis and CV calcification—the so called osteoporosis–vascular calcification paradox [64,65]. However, current evidence suggests that improving bone mineral density does not alter CV calcification. 

**Table 2:** Novel therapeutic strategies that attenuate CV calcification in non-transgenic animal CKD models.

Treatment	Substance	Dosis	Application	Experimental Model	Species, Strain	Ref.
Bisphospho-nate	Etidronate	5 or 10 mg/kg	s.c., daily, 3 weeks	5/6 nephrectomy	Wistar rat	[54]
Vitamin K	Mena-quinone-7	50 µg/kg	Oral gavage, daily 4 weeks	Adenine diet	Sprague-Dawley rat	[66]
Omega-3 fatty acid	Eicosapenta-enoic acid	300 mg/kg	Oral gavage, daily 4 weeks	Adenine diet	Sprague-Dawley rat	[66]
Vitamin D receptor agonist	Calcitriol Paricalcitol	30 ng/kg 100 or 300 ng/kg	i.p., 3 times/week, 3 weeks	5/6 nephrectomy	DBA/2J mouse	[26]
Dietary supplement	Magnesium	0.1–1.1%	Food intake, 14 days	5/6 nephrectomy	Wistar rat	[67]
Dietary supplement	Magnesium	3%	Food intake, 7 weeks	5/6 nephrectomy	Non-agouti mouse	[68]
Hexasodium salt	SNF472	50 mg/kg	i.v., daily, 19 days	Adenine diet	Wistar rat	[20]

S.c: subcutaneous; i.p.: intraperitoneal; i.v.: intravenous; Ref: Reference.

### 4.2. Vitamin K

Vitamin K is a cofactor for post-translational γ-carboxylation of calcification inhibitors and activators that plays a role in mineralization and osteogenic differentiation of vascular smooth muscle cells. More importantly, in CKD, vitamin K serves for the carboxylation of the calcification inhibitor MGP and the vitamin K-dependent calcium binder osteocalcin [69,70]. Vitamin K deficiency causes reduced carboxylation of uncarboxylated MGP (ucMGP) to carboxylated MGP (cMGP). Therefore, the inhibiting effect of cMGP is attenuated. The inactive form of ucMGP, which is dephosphorylated (dp-ucMGP), can be measured as a representative for the vitamin K status. In medial calcification (Mönckeberg’s sclerosis), which is associated with renal disease, MGP is expressed in all calcified areas in human tissue samples [71]. There are two naturally occurring vitamers of vitamin K, that differ in bioavailability and distribution in the human body: vitamin K1 (phylloquinone) and vitamin K2 (menaquinone, MK). While the former is mainly retained in the liver to serve as a cofactor for the carboxylation of clotting factors, circulating vitamin K2 is available for the extrahepatic tissue and the vascular system [72] and thereby is more capable of acting in the vascular calcification process [70]. Due to its bioavailability, the vitamer MK-7 is mainly used in clinical trials [72]. A single-MK-7 treatment, as well as the combination of MK-7 and eicosapentaenoic, reduced the development of vascular calcification in an experimental model of adenine-induced CKD rats (Table 2) [66]. 

HD patients show low vitamin K intake, accompanied by increased levels of serum dp-ucMGP [73]. One explanation for this result could be the recommendation for CKD patients to avoid phosphate- and potassium-rich food, which often contains vitamin K [74]. Consequently, vitamin K deficiency increases the risk for vascular calcification in already calcification-prone CKD patients. A prospective cohort study of patients in CKD stages 4 to 5D revealed a positive correlation between serum dp-ucMGP levels and aortic calcification (Table 3) [75]. All-cause mortality was higher in patients with dp-ucMGP levels above the median [75]. HD patients did not reveal a positive correlation of the dp-ucMGP levels with the extent of vascular calcification [75]. After adjustment, low dp-cMGP levels were associated with a higher all-cause and CV mortality risk [75]. In a cohort with stable KTR, all-cause mortality was increased in patients in the highest dp-ucMGP quartile compared to the lowest quartile, after adjustment and exclusion of vitamin K antagonists [76]. 

In vitro studies showed the binding of vitamin K by PB [77]. Therefore, PB inhibit the gastrointestinal uptake of vitamin K2, thus aggravating vitamin K deficiency in CKD patients [78]. In a study, calcium acetate and magnesium carbonate bound to vitamin K2, independent of the presence of phosphate, while sevelamer carbonate did not bind to vitamin K2 in vitro [64]. This could be one additional explanation as to why non-calcium-based PB are favored in the studies mentioned above. Interestingly, the non-calcium-based PB lanthanum bound to vitamin K2 only in the absence of phosphate [78]. In order to investigate the effect of PB on vitamin K deficiency in vivo, a cross-sectional study with HD patients, patients with peritoneal dialysis, and KTR was performed. Dp-ucMGP levels were significantly lower in KTR compared to dialysis patients. No association between the use of any PB and dp-ucMGP was observed, while sevelamer monotherapy was associated with elevated dp-ucMGP levels [79]. This evidence does not fit the in vitro observation that sevelamer did not bind to vitamin K2 [64]. The clinical relevance of the influence of PB on vitamin K deficiency remains unclear. 

Clinical interventional trials investigated the effect of vitamin K2 supplementation in CKD patients with vitamin K deficiency (Table 4). In HD patients, MK-7 treatment reduced dp-ucMGP levels, while dp-cMGP did not alter them [75,80,81]. In CKD patients stage 3–5, a combined treatment of MK-7 and vitamin D reduced dp-ucMGP levels and carotid–intima–media thickness, compared to vitamin D therapy only [82]. The CAC score was increased in both groups. Due to the growing interest in vitamin K biology and the role in preventing CV calcification, ongoing randomized controlled trials on vitamin K supplementation in CKD patients are taking place [82]; according to the status update on clinicaltrials.gov, results have yet to be published.

### 4.3. Vitamin D

Vitamin D deficiency and sHPT are common comorbidities in progressive CKD stages. Vitamin D application lowers PTH levels in the body. TheKidney Disease: Improving Global Outcomes KDIGO guideline from 2017 recommends vitamin D analogs for both CKD pre-dialysis patients stage 4 and 5 and dialysis patients with sHPT [11]. 

In a mouse model of CKD with electrocoagulation of the right renal cortex and left nephrectomy, treatment with the vitamin D receptor agonists calcitriol and paricalcitol prevented calcification (Table 2) [26]. A meta-analysis of 20 observational studies revealed an association of vitamin D supplementation in pre-dialysis and HD patients with decreased all-cause and CV mortality [84]. The association between vitamin D deficiency and endothelial dysfunction supports the hypothesis that vitamin D supplementation could attenuate vascular calcification in CKD patients [85]. Therefore, interventional studies investigated the effect of vitamin D analogs on arterial stiffness. A double-blind RCT compared the effect of calcifediol (25-hydroxyvitamin D3) and calcitriol (1,25-dihydroxyvitamin D3) to placebo by analyzing pulse wave velocity (PWV) as a parameter for vascular stiffness [86]. PWV was decreased in the calcifediol group, while it stagnated in the calcitriol group and was increased in the placebo control. Furthermore, cholecalciferol improved vascular stiffness in pre-dialysis patients compared to placebo, suggesting a beneficial effect of cholecalciferol on endothelial function [87]. However, treatment with cholecalciferol did not significantly attenuate CAC in CKD [88]. Evidence for a beneficial effect of vitamin D supplementation on CV calcification progression remains uncertain. The informative value is also limited by the use of different vitamin D analogs and dosages. Further RCT are necessary to evaluate the potential of vitamin D supplementation in CKD. Findings demonstrated a vitamin D level decline prior to the occurrence of changes in PTH and phosphate. Therefore, earlier vitamin D supplementation should be considered in patients without sHPT [89].

### 4.4. Magnesium

Magnesium is a micronutrient with various functions in the body. In vitro studies revealed an inhibiting role of magnesium in phosphate-induced calcification [90]. Dietary magnesium supplementation reduced and reversed vascular calcification in five-sixths nephrectomized rats (Table 2) [67]. These findings were supported by Kaesler et al., showing that magnesium treatment reduces vascular calcification in five-sixths nephrectomized mice (Table 2) [68]. 

A negative association of serum magnesium with vascular calcification was shown in CKD patients [91]. In a meta-analysis encompassing 532,979 patients from 19 prospective cohort studies of the general population, serum magnesium as well as dietary magnesium intake was inversely associated with the risk of CV events [92]. This observation was confirmed in different observational studies in HD and peritoneal dialysis patients. Lower serum magnesium levels were associated with higher all-cause and CV mortality [93,94,95,96]. However, hypomagnesemia was not an independent predictor for mortality in end-stage renal disease [93,96]. CAC and vessel stiffness occurred in patients with high magnesium serum levels [97]. Although these observations encourage the assumption that magnesium supplementation might attenuate vascular calcification in CKD, few interventional studies have been performed. In HD patients, magnesium treatment reduced carotid intima–media thickness compared to placebo control [98]. Carotid intima–media thickness was also improved in a small trial with magnesium citrate, compared to treatment with the PB calcium acetate [99]. In the ongoing MAGiCAL-CKD trial, pre-dialysis patients (*n* = 250) are treated with 360 mg/day magnesium hydroxide for one year. The change in CAC will be evaluated by CT scans [100]. Results of this study might provide new evidence concerning the role of magnesium in the prevention of CV calcification in CKD. 

### 4.5. Hexasodium Salt of Myo-Inositol Hexaphosphate

A novel therapeutic option is the hexasodium salt of myo-inositol hexaphosphate SNF472, a potent calcification inhibitor in vitro [20]. SNF472 binds to the growth sites of hydroxyapatite crystals, the main constituent part of calcification deposits, thereby reducing the progression of ectopic calcification [20]. SNF472 inhibited CV calcification in adenine-induced CKD rats by up to 90% (Table 2) [20]. In ex vivo analysis using plasma from HD patients, hydroxyapatite crystallization potential was reduced by SNF472 [101,102]. The first phase 2 study CaLIPSO with 274 HD patients demonstrated attenuated progression of CAC and aortic valve calcification compared to placebo control, after 52 weeks of SNF472 treatment [21].

## 5. Promising Treatments of CV Calcification in Experimental CKD Models

Opportunities for renal transplantation are low, and many patients suffer from progressive CKD and its comorbidities. Existing drug therapies offer no adequate solution to treat/prevent CV calcification in CKD patients. In experimental non-transgenic CKD models, new promising therapeutic interventions and potential drug targets to decrease CKD-induced calcification and prevent or reverse pathophysiological complications have recently been shown. The isoflavonoid compound puerarin, found in the root of *Pueraria lobata*, has anti-inflammatory effects [103] and inhibited calcification in mouse vascular smooth muscle cells [76] and five-sixths nephrectomized rats (Table 5) [104]. 

PPARγ plays an important role in CVD and is closely connected to atherosclerosis [105,106]. Rosiglitazol, a PPARγ agonist, reduced vascular calcification in five-sixths nephrectomized mice (Table 5) [107]. Another potential drug target for CV calcification in CKD could be the nuclear factor kappa-light-chain-enhancer of activated B cells (NF-κB), which is active in calcified vessels [108]. The NF-κB inhibitors tempol and triptolide reduced vascular calcification in an adenine-induced CKD mouse model [109], as well as in adenine-induced CKD rats (Table 5) [110]. Further, different studies have shown that MR signaling can promote CV calcification [92]. Blockage of MR is increasingly applied as a therapy for improvement of CV outcomes in CKD, diabetes mellitus, hypertension, and heart failure. The MR antagonist spironolactone improved CV outcomes in patients with heart diseases [92] and inhibited dose-dependent vascular calcification and kidney damage in adenine-induced CKD rats (Table 5) [111]. 

**Table 5:** Potential therapeutic strategies that attenuate CV calcification in non-transgenic animal CKD models.

Treatment	Substance	Dosis	Application	Experimental Model	Species, Strain	Ref.
Isoflavonoid	Puerarin	400 mg/kg	Oral gavage, daily; 4 weeks	5/6 nephrectomy	Sprague-Dawley rat	[104]
PPARγ agonist	Rosiglitazol	10 mg/kg	Oral gavage, daily; 12 weeks	5/6 nephrectomy	DBA/2J mouse	[107]
NF-κB inhibitor	Tempol	3 mmol/L	Drinking water; 10 weeks	Adenine diet	DBA/2J mouse	[109]
NF-κB inhibitor	Tempol	3 mmol/L	Drinking water; 6 weeks	Adenine diet	Sprague-Dawley rat	[110]
NF-κB inhibitor	Triptolide	70 µg/kg	i.p., daily; 10 weeks	Adenine diet	DBA/2J mouse	[109]
MR antagonist	Spirono-lactone	100 mg/kg	Food intake, daily; 2 weeks	Adenine diet	Sprague-Dawley rat	[111]

MR: mineralocorticoid.

## 6. Potential Diagnostic Tools for CV Calcification in CKD

### 6.1. Development of the T_50_ Assay

Circulating biomarkers associated with progression of vascular calcification and mortality in CKD patients lack predictive value. For example, serum levels of fetuin-A and osteoprotegerin positively correlate with mortality of dialysis patients, and soluble klotho is associated with aortic calcification progression [112,113]. In 2012, Pasch et al. introduced a novel concept for the risk assessment for CKD patients. The T_50_ assay is a measure of the propensity for calcification in blood serum [114], based on the time-dependent shape change of calcium-phosphate precipitation particles. Colloidal spherical-shaped primary calciprotein particles (CPP) convert to crystalline secondary CPPs with radial growth of crystalline needles [115]. Nephelometry allows the determination of the transition step from primary to secondary CPPs. The amount of precipitation depends on the capacity of serum to inhibit this process by calcification inhibitors like fetuin-A. In this assay, the patient’s serum is supersaturated by adding 6 mM phosphate and 10 mM calcium to accelerate precipitation. This allows the analysis of the half-maximal transition time (T_50_). Higher T_50_ values reflect longer transition times, thereby less propensity for calcification. A potential clinical use needs to be evaluated [114].

### 6.2. Clinical Association

An association of shorter T_50_ times with increased all-cause and CV mortality, as well as CV events, could be demonstrated in pre-dialysis CKD patients, HD patients, and KTR (Table 6) [76,107,116,117,118]. Aortic pulse wave velocity (APWV), as a quantification tool of progressive arterial stiffness and vascular calcification, showed conflicting results in association with T_50_ [116,118]. In KTR, baseline APWV was not associated with T_50_ values [118], while an association of lower T_50_ values with increasing APWV was found in patients with CKD stage 3 and 4 [116]. T_50_ values are not associated with CAC prevalence but rather with greater CAC severity (Table 6) [97]. Further investigations considering clinical parameters that represent the progression of vascular calcification should be made to estimate the predictive value of T_50_ with respect to calcification in CKD patients. 

## 7. Outlook

Pharmaceutical treatments currently applied in clinical routine offer no adequate solution to treating or preventing CV calcification in CKD. Currently, we have no clear evidence that direct targeting CV calcification leads to an improvement in CV outcomes in CKD and ESRD patients. Still, vitamin K supplementation diminished the progression of aortic valve calcification and subsequently affected the cardiac and clinical outcomes in CVD patients without CKD [119], giving hope that future developments will yield the must needed treatment option to reduce CV risk in CKD patients. In experimenal CKD rodent models, new promising therapeutic interventions and potential drug targets to decrease CKD-induced calcification and prevent or reverse pathophysiological CV complications have recently been shown. However, no single animal model thoroughly reproduces the complexity of CV calcification in CKD and all attendant comorbidities. For this reason, it is essential to agree on a consistent animal model within this research area to maintain comparability. 

## Figures and Tables

**Table 1:** Therapeutic strategies that attenuate CV calcification in non-transgenic animal CKD models.

Treatment	Substance	Dosis	Medication	Experimental Model	Species, Strain	Ref.
Phosphate binder	Sevelamer	750 mg/kg	Daily oral gavage, 4 weeks	Adenine diet	Wistar rat	[32]
Phosphate binder	Sevelamer	3%	Diet, 6 months	5/6 nephrectomy	Sprague-Dawley rat	[31]
Phosphate binder	CaMg	185 mg/kg	Daily oral gavage, 6 weeks	Adenine diet	Wistar rat	[33]
Calcimimetic	Cinacalcet	10 mg/kg	Daily oral gavage, 12 weeks	Adenine diet	Wistar rat	[38]

CaMg: acetate/magnesium carbonate; Ref: Reference.

**Table 3:** Observational studies investigating the role of vitamin K in CKD.

Patients	Follow-up	Main Results	Ref.
CKD stages 4 to 5D (*n* = 107)	2.2 years	dp-ucMGP: positive association with progressive CKD stages and increased all-cause mortality	[75]
HD patients (*n* = 188)	3 years	- 6.5-fold elevated dp-ucMGP - dp-cMGP associated with increased all-cause and CV mortality	[75]
KTR (*n* = 518)	9.8 years	dp-ucMGP: association with increased all-cause mortality	[76]

HD: hemodialysis; KTR: kidney transplant recipients; dp-ucMGP: dephosphorylated-uncarboxylated matrix Gla protein; dp-cMGP: dephosphorylated-carboxylated matrix Gla protein.

**Table 4:** Interventional studies investigating the effect of vitamin K in CKD.

Patients	Treatment	Study Design	Main Results	Ref.
CKD stage 3–5 (*n* = 42)	90 μg/d MK-7 + 10 μg/d cholecalciferol, or 10 μg/d cholecalciferol (control), 38.5 weeks	Prospective, randomized, double-blind	Decrease of dp-ucMGP, smaller increase of CAC and CCA-IMT compared to control	[83]
HD patients (*n* = 50)	360 μg/d MK-7, 4 weeks	Prospective, pre-post intervention clinical trial	86% decrease of dp-ucMGP	[80]
HD patients (*n* = 17)	135 μg/d MK-7, 6 weeks	Interventional pilot study	Decrease of dp-ucMGP but not dp-cMGP	[75]
HD patients (*n* = 53), Healthy controls (*n* = 50)	45, 135, 360 μg/d MK-7, 6 weeks	Interventional, randomized, non-placebo-controlled trial	Dose-dependent decrease of dp-ucMGP	[81]

MK-7: menaquinone-7 (vitamin K2); CAC: coronary artery calcification; Vit.K: vitamin K2; CCA–IMT: common carotid artery–intima media thickness.

**Table 6:** Clinical assessment of calcification propensity based on half-maximal transition time (T_50_) in CKD patients.

Patients	Mean/Median T_50_ (Baseline)	Follow up, Years	Findings	Ref.
CKD stages 2 to 4 (*n* = 1274), In follow up *n* = 780	Median: 321 min	3.2	Association of low T50 with increased CAC prevalence and progression	[97]
CKD stages 3 and 4 (*n* = 184)	Mean: 329 ± 95 min	5.3	Association of low T50 with increased all-cause mortality and APWV	[116]
HD patients (*n* = 2785), control group (*n* = 1366)	Mean: 212 min (10th–90th percentile: 109–328 min)	1.7	Association of low T50 with increased all-cause mortality and CVD	[117]
HD patients (*n* = 188)	Mean: 246 ± 64 min	3.7	Association of low T50 and T50 decline with all-cause and CV mortality	[117]
KTR (*n* = 699)	Mean: 286 ± 62 min	3.1	Association of low T50 with increased all-cause and CV mortality and graft failure	[76]
KTR (*n* = 433)	Mean: 340 ± 70 min	3.7	Association of low T50 with increased CVD event risk	[107]
KTR during 10 weeks after transplantation (*n* = 1435), Follow-up: APWV after 1 year (*n* = 589)	Median: 188 min (25th–75th percentile: 139–248 min)	5.1	Association of low T50 with increased all-cause and CV mortality APWV not associated with T50 baseline	[118]

APWV: aortic pulse wave velocity.
